# Comparative Study of Newer and Established Methods of Diagnosing Coccidioidal Meningitis

**DOI:** 10.3390/jof6030125

**Published:** 2020-08-04

**Authors:** David A. Stevens, Marife Martinez, Gabriele Sass, Demosthenes Pappagianis, Brian Doherty, Hannah Kutsche, Meredith McGuire

**Affiliations:** 1 California Institute for Medical Research, 2260 Clove Drive, San Jose, CA 95128, USA; mmartinez@cimr.org (M.M.); gabriele.sass@cimr.org (G.S.); 2 Division of Infectious Diseases and Geographic Medicine, Stanford University School of Medicine, Stanford, CA 94305, USA; 3 Department of Medical Microbiology, University of California School of Medicine, Davis, CA 95616, USA; dpappagianis@ucdavis.edu; 4 IMMY, Inc., 2701 Corporate Center Drive, Norman, OK 73069, USA; brian-doherty@immy.com (B.D.); hannah-kutsche@immy.com (H.K.); meredith-mcguire@immy.com (M.M.)

**Keywords:** *Coccidioides*, coccidioidomycosis, meningitis, enzyme immunoassay, lateral flow assay, fungal diagnosis

## Abstract

Meningitis is the most devastating form of coccidioidomycosis. A convenient, rapid diagnostic method could result in early treatment and avoid many meningitis complications. We studied cerebrospinal fluid (CSF) samples in patients with documented coccidioidal meningitis, and controls, with complement fixation (CF), immunodiffusion (ID) (the “classical” assays), lateral flow assays (LFA; one-strip and two-strip), and two enzyme immunoassays (EIA). The two-strip LFA and EIAs not only enabled separate testing for IgG and IgM antibodies separately, but also could aggregate results for each method. CF with ID or the aggregate use of IgG and IgM tests were considered optimal test uses. LFAs and EIAs were evaluated at 1:21 and 1:441 dilutions of specimens. All assays were compared to true patient status. With 49 patient specimens and 40 controls, this is the largest comparative study of CSF coccidioidal diagnostics. Sensitivity of these tests ranged from 71–95% and specificity 90–100%. IgM assays were less sensitive. Assays at 1:441 were similarly specific but less sensitive, suggesting that serial dilutions of samples could result in assays yielding titers. Agreement of positive results on cases was 87–100%. When kits are available, hospital laboratories in endemic areas can perform testing. LFA assays do not require a laboratory, are simple to use, and give rapid results, potentially even at the bedside.

## 1. Introduction

Of all the complications of disseminated coccidioidomycosis, the most lethal is meningitis; it is estimated that there are 200–500 new cases of this complication per year [1]. Treatment with oral antifungals requires lifetime administration to suppress recurrences [2], and in many cases intrathecal (and neurotoxic) therapy is required to stop progression [3]. Because of the many complications, which include hydrocephalus, vasculitis, cerebral or spinal cord infarction, arachnoiditis, cranial nerve palsy, syringomyelia, transverse myelitis, cord compression, paralyses, parenchymal abscesses, and seizures, we deem it essential to begin treatment as early as possible, before the pathologic processes have advanced or become irreversible. Early diagnosis is thus very desirable.

Culture of cerebrospinal fluid (CSF) for *Coccidioides*, even in active and untreated cases, is positive in a minority of specimens, presumably relating to the focalization of disease to the meninges themselves and the absence of fungal multiplication in CSF. The classical method for diagnosis is the detection of anti-coccidioidal antibodies in the CSF [4]. The antibodies that react in coccidioidal assays are IgG and IgM antibodies; IgM is usually detected early in the course and IgG persists during disease activity and beyond. IgG antibodies appear directed against the chitinase enzyme of this fungus (this antigen is often referred to as IDCF, detected in complement fixation (CF) or immunodiffusion (ID)), and IgM antibodies to a polysaccharide-containing fungal antigen, incorporating the coccidioidal beta-glucosidase (this antigen is often referred to as IDTP, detected in tube precipitation assays or ID). IDCF [5] and IDTP [6] have been defined at a molecular level.

In the present study, we compared lateral flow assay—a new convenient and rapid method used for CSF antibody detection—to older methods and other assays that have been applied to detection of fungal products in the CSF. All comparisons, including “classical tests” (CF and ID), were made with true disease status (defined in Section 2.1).

## 2. Methods

### 2.1. Populations Studied 

Patients considered to have coccidioidal meningitis [7] had clinical meningitis, including a CSF that indicated inflammation (i.e., pleocytosis, an elevated CSF protein, and, in some cases, depressed CSF glucose) and coccidioidal antibody positive or culture positive CSF specimens (at any time in their course, though not necessarily the specimen obtained for the present study), coccidioidal antibody in serum or coccidioidal culture from another body site, as per previously established guidelines [8]. All patients received therapy for meningeal disease, some (with refractory disease) with intrathecal amphotericin B [3], and, as previous studies indicated therapy with azoles requires lifelong administration [2], received lifelong azoles. CSF specimens (3 ventricular and the remainder lumbar; 35 patients) were collected over 19 years and available for testing. The control patients had a lumbar puncture in the outpatient department or as inpatients, because of suspected microbial meningitis on clinical grounds, with meningismus, a CSF usually indicating inflammation, and with no clinical evidence of CNS or systemic fungal diagnosis at the time of the lumbar puncture or on subsequent follow-up. Most of these patients were discharged with a diagnosis of viral or bacterial meningitis. We utilized the results of tests previously requested by the patients’ clinicians (CF and ID testing were done as part of the patients’ management) or residuals of previous completed tests (including drug assays). Consent for assays for research and education purposes on the residuals, without use of patient identifiers, had been given on a standard form at the time of patient registration [7]. Testing personnel were blinded to the diagnosis or patient identifiers prior to performing the study. Specimens were stored at −80 °C and thawed to room temperature before analysis. In some instances, there was insufficient quantity of a specimen to allow all the assays that were included in the present study. 

### 2.2. Assays

#### 2.2.1. Classical Methods

For decades, the assays for CF and precipitating antibodies [4] in serum, CSF, and other body fluids were the mainstay of coccidioidomycosis diagnosis. Herein, we regard this as “the classical test” for comparative purposes. With serial dilution of specimens, in either CF or ID to detect precipitating antibodies, a titer can be derived, designating the highest dilution to give a positive assay result. The titer is the last dilution with a 3+ (out of 4+) complement fixation reaction. It should be noted that this testing starts with an undiluted specimen (in comparison to the tests to be described that assayed at a 1:21 starting dilution). Testing by the classical method proceeded in the following manner [4]: presence of CF antibodies was assayed first. If the result was negative and if there was a sufficient specimen, then it was concentrated and tested by ID. A positive test in ID was then considered antibody-positive. ID can also be used for anti-complementary sera, as that interferes with CF testing. If the serum has a high CF titer, anti-complementarity present in low concentration can be removed as an interfering factor by diluting in titering, beyond the anticomplementarity. 

#### 2.2.2. Lateral Flow Assays (LFA) 

*Coccidioides* antibody testing was also performed using the IMMY (Norman, OK, USA) sōna Valley Fever Dx IgG/IgM LFA (Ref# CAB2003), which is not yet FDA-approved. This test is hereafter referred to as the two-strip test. This assay consists of two test strips. One strip is used for the detection of IgG antibodies and the other is for the detection of IgM. 

In the recently released sōna *Coccidioides* Ab LFA (Ref# CTA2003), hereafter referred to as the one-strip test, the assay detects both IgG and IgM antibodies of a coccidioidal antigen on a single test line and does not distinguish between the two immunoglobulins. 

The mechanics of the lateral flow design allows for a more rapid detection of anti-coccidioidal antibodies. The design of both test kits consists of antibody-binding proteins conjugated to colloidal gold nanoparticles that, upon interacting with a diluted patient specimen in wells, will bind and migrate up the test strip. The LFA strips were inserted into the well and the assays scored 30 min later. The test lines consist of a blend of diagnostically relevant antigens bound to nitrocellulose, including IDCF and IDTP, combined on the one-strip test or separated on the two-strip test. If the patient has antibodies against these antigens, the antibody-gold complex will bind and cease to migrate, eventually forming a red test line. Residual antibody-gold complexes will bind further up the strip at a control point that contains anti-human antibodies, to give a line providing evidence of proper wicking and binding. Patients without anti-coccidioidal antibodies will not bind to the test line and only to the control line, which indicates a negative test. Patients with a test and control line are considered positive. No lines or only test line showing are indicative of an invalid test. Diagrams of the tests in use are available online and included in test kits. In the present study, four readers—two experienced and two novice—independently read each test strip for the appearance of the lines. We adopted a very conservative posture in an attempt to consider how these tests would be used in clinical practice, even taking one discrepant result among these four observer tests as an indeterminate result. For purposes of this study, observers were asked to record positive results as “clear positives” or “weak positives”. It should be noted that during the study, only serum was FDA-approved for the LFAs (the one-strip was waived by the FDA for serum).

#### 2.2.3. Enzyme Immunoassays (EIAs)

The basic mechanics of EIAs are well known [9]. The IMMY clarus *Coccidioides* antibody ELISA (Ref# CAB102) was evaluated for the detection of coccidioidal antibodies in CSF specimens. The assay is FDA approved for the detection of coccidioidal antibodies in serum specimens. It should be noted that serum is the only approved test specimen at this time. It was used according to the manufacturer’s instructions, modified for CSF testing, as now described.

The assay consists of two separate plates, one for IgG and one for IgM; each has the diagnostically relevant antigens adsorbed to each 96-well plate. Briefly stated, 100 µL of diluted patient specimen was incubated for a 30-min primary incubation at room temperature followed by three, 200 µL washes of the plate to remove unbound material. An additional 30-min, room temperature incubation was performed with 100 µL of horse radish peroxidase-conjugated secondary antibody detection reagent, followed by a wash step. Finally, 100 µL of 3, 3′, 5, 5′ tetramethylbenzidine color developer was applied to the wells and incubated for 10 min. at room temperature, followed by 100 µL of the acid stop solution provided in the kit. The optical density of each well is read by a spectrophotometer at 450 nm and 630 nm and compared to a calibrator to determine the optimal cutoff. 

Further data analysis was performed using MedCalc software [10], generating dot plots and receiver-operator curves. Preliminary studies with CSF, on a subset of samples, indicated the breakpoint for CSF in the IMMY EIA should be 0.3 units as the cutoff for positivity. Using this value, the receiver operating characteristic curve had an area under the curve of 0.937 for IgG, and 0.840 for IgM, indicating good levels of discrimination and accuracy (Supplementary Figure S1). This program was also used to calculate r^2^, the correlation coefficient [10], to compare tests giving quantitative results.

Meridian (Cincinnati, OH, USA) Premier *Coccidioides* antibody ELISA (Ref# 603096) is FDA approved for the use of CSF or serum specimens in the detection of coccidioidal antibodies. The assay was performed according to the package insert, with a provided diluent. Separate wells are used for the detection of IgG and IgM. Patient specimens were diluted in a 96 well plate, and assayed according to the manufacturer’s instructions for use. The plates were read at 450 nm with a 630 nm reference wavelength using a BioTek (Winooski, VT, USA) ELX800 plate reader. Data was interpreted using the package insert criteria for CSF specimens, a 0.2 absorbance unit threshold for positivity. This test also has an indeterminate result, 0.15–0.199 absorbance units.

We studied the utility of the LFA and EIA assays with CSF specimens at two levels of patient dilution. CSF specimens were diluted in each respective kit’s specimen diluent using flat-bottomed 96 well plates to two arbitrarily chosen dilutions, 1:21 and 1:441, for direct comparison purposes, and the results evaluated separately. The 1:21 dilution was chosen as it is the dilution utilized in the Meridian EIA assay, and the 1:441 dilution as it is the one used in testing of serum specimens in the IMMY assay. Each specimen dilution was tested in the assay according to the kit instructions for use. Where there was independent testing of IgG and IgM, a positive in either test (the other negative, or indeterminate as defined), the result was considered positive. If one test was indeterminate, and the other negative, the result was considered indeterminate.

#### 2.2.4. Antigen and Nucleic Acid Study

The assay for CSF beta-D-glucan (BDG) relied on the Fungitell kit (Associates of Cape Cod, Falmouth, MA, USA). The BDG data included here, for comparative purposes with present results on the same specimens, were previously described [7], in a coccidioidal meningitis study. A positive is 31 pg/mL.

*PCR.* PCR data (3 samples), for comparative purposes with present results on the same specimens, was derived from a prior study [11]. 

#### 2.2.5. Rabbit CSFs

Residual CSFs taken from rabbits as part of therapeutic studies of coccidioidal meningitis, as previously described [7,12,13], were studied by 1-strip LFAs.

## 3. Results

### 3.1. Assays in Diagnosing Coccidioidal Meningitis, LFAs

At a 1:21 dilution the one-strip LFA had a sensitivity of 95% and a specificity of 100%, a positive predictive value (PPV) of 100% and a negative predictive value (NPV) of 86% (Table 1). Six (of 60) indeterminate results, as defined, were excluded; 5 of these were due to some observers recording the test as negative and others as a weak positive. The 1:441 dilution was less sensitive (69%) but as specific (100%). 

The 2-strip test, IgG assay had a sensitivity of 92%, specificity 100%, PPV 100%, NPV 91% (Table 2), with 1:441 again less sensitive (70%). The 2-strip IgM test was 53% sensitive, 100% specific, and the 1:441 assay again less sensitive, 20%. The lesser results with IgM are consistent with the usual dominance of IgG antibodies found in CSF. 

When considering testing with both strips at 1:21, the combined sensitivity resulting from both tests was 92%, specificity 100% (PPV 100%, NPV 91%; Table 3), and at 1:441, less sensitive, 70%. Ten (of 77) 1:21 results were declared indeterminate, as defined here; 7 of these were due to the “negative vs. weak positive” discrepancies- thus again, discrepancies were very rarely between clear positive and negative readings. 

In summary then, with the 1-strip, or testing IgG and IgM in the 2-strip test, the results were very similar (slightly improved NPV in the latter), and highly efficacious in use for diagnosing coccidioidal meningitis.

### 3.2. Classical Methods

The classical test, assaying CF testing alone, was also efficacious, less sensitive (71%), but as specific (100%). The PPV was 100% and the NPV 69% (Table 4a). When ID testing could also be performed, and ID results available, the combined CF+ID results then increased the sensitivity to 78% (specificity 100%), with PPV 100% and NPV 74% (Table 4b). More information about CF and ID results is given below, in the section “Correlation of tests”.

### 3.3. EIAs

With the application of the IMMY EIA to specimens, studying IgG only, the sensitivity was 90%, specificity 93% (PPV 94%, NPV 90%). With the IgM assay only, the sensitivity dropped to 49%, though specificity remained at 93%. When the results were combined (i.e., considering a test of either IgG or IgM positive on any specimen as positive), the sensitivity was 94%, and specificity 90% (PPV 92%, NPV 92%) (Table 5). 

In none of these assays was there an indeterminate result. Thus in this IMMY EIA assay, IgM testing added little to the efficacy of IgG testing alone of CSF. In considering IgG or IgM alone, or the use of both tests, the 1:441 dilution test was less sensitive, 15–75% among those 3 comparisons, but specificity remained high (98–100%).

With the Meridian EIA, tested 1:21, studying IgG only, the sensitivity (71%) was less than the IMMY EIA, though specificity was 100%. With IgM assay, the sensitivity was much less than with Meridian IgG, or IgM in IMMY EIA, only 7% (specificity, 100%). When comparing the utility of both the IgG and IgM results, as discussed above re the IMMY test, the sensitivity (71%) was inferior to the IMMY EIA, though specificity was 100% (PPV 100%, NPV 68%). One of 68 of all these Meridian assays was indeterminate (as defined by the kit).

### 3.4. Summary: Test Correlations

For summary and ease of comparison, the results of all tests, used in their optimal fashion, is repeated in Table 6.

### 3.5. Percent Agreement between Tests

The percent agreement between all tests, on positives or negatives, was very good, with the least agreement between BDG antigen results and the antibody assays. These comparisons will be affected by the differences in sensitivity between tests, already described.

For example, considering all tests used in their optimal (CF + ID; for other tests, combining the results of IgG and IgM testing), the agreements among positive assays for each of the 5 antibody tests with each other ranged from 87–100%, with all but one being ≥90% (Table 7). Agreements on positives of the 5 antibody tests with the BDG assay ranged from 68–90%.

### 3.6. Correlations of Tests

The correlations between tests are seen in the comparison of the assay results (first Results section), and also the above section on agreements between tests. Two tests, the CF and the BDG test, give quantitative results, beyond only positive or negative. In a previous study [7], there was no correlation found between the BDG CSF antigen titer test and the CSF CF antibody titer. In examination of the present results, there was also no impressive correlation with CSF BDG antigen titer and 1-strip LFA, 2-strip LFA, IMMY EIA, or Meridian EIA antibody tests outcome either; the r^2^ correlation coefficients ranged from 0.12 to 0.21. 

On the other hand, with both the IMMY EIA and the Meridian EIA, the only negative CSF EIA with each test that had CF titration, had CF titers of only 1:2. The r^2^, with CSF CF for the IMMY IgG EIA was 0.38, and for the Meridian EIA 0.25. Overall, the CSF CF positive titers in the present study were: undiluted only, 4; 1:2, 8; 1:4, 2; 1:8, 6; 1:16, 2.

Two patients had serum drawn the same day as a CSF sample was available. Both samples from both were positive in IMMY EIA, but there was insufficient testing by other assays to draw conclusions about concordance between the 2 samples.

Of the CSFs from cases in the present study, we obtained the ID results for 2 that were CF negative and 2 that were CF positive (titers 1:2), and in these 4 the ID results were positive. In another case, the quantity was insufficient for a CF test undiluted, a 1:2 dilution was CF negative, and the ID was positive. 

The CSF CF negative cases were of particular interest, as these might reflect CSFs with the very lowest antibody concentrations. The 2 CF negative, ID positive specimens above were positive in both LFAs, IMMY EIA, and BDG, and one of two Meridian EIA positive. 

In addition to those, there were 7 others CF negative. All but one appeared to be on therapy, and thus their disease possibly stabilized or in remission. One of these was a ventricular CSF, this entity to be discussed later. Of the other 6, 2 were ID negative as well and 4 had had no ID done (apparently owing to inadequate sample). Of these 6 CF negatives, 5 were positive in the 1-strip LFA and the IMMY EIA, 3 of 4 were positive in the 2-strip LFA (2 indeterminate), 5 of 5 tested were BDG positive, and only 1 of 6 positive in the Meridian EIA. This suggests a weakness in Meridian EIA when antibody titers are low. In one of the 6 (this one positive in the 2 LFAs and IMMY EIA, negative in Meridian EIA; not BDG tested), we were able to find a record that a second CSF (unavailable to us), tested 1 mo. later, had turned CF positive, indicating those LFA and IMMY EIA positive assays on our earlier specimen had detected a case in the early stage. 

### 3.7. PCR

Of cases that were studied with PCR, and negative, 2 of 3 could be studied presently. One was a ventricular specimen, these discussed below. The other was negative in CF, IMMY and Meridian EIA testing, indeterminate in the 2-strip LFA, and only positive in the 1-strip and BDG assays.

### 3.8. Ventricular Specimens

Ventricular CSF specimens represent an especially difficult instance in CSF testing, because, unless there is ventriculitis, the ventricles have disproportionately less inflammation than concurrent lumbar specimens, and because of the dilution in ventricular volume, detecting antibody and antigen would also be more difficult. Three specimens were included in the present study, but not all the specimens could be tested by all assays. Two were CF negative, but one of these was ID positive (also PCR negative). Two of 3 were Meridian EIA positive, 2 of 2 were BDG positive, and all 3 were positive by 1-strip LFA, 2-strip LFA and IMMY EIA.

### 3.9. Serial Specimens

Multiple lumbar CSF specimens were available from 7 patients. Of patients with samples obtained 1 year or less apart, their assays (CF, 1- and 2-strip LFAs, IMMY and Meridian EIAs, and BDG) were generally very consistent over time. However, samples were obtained from 2 patients separated by 7 years on treatment, and in both patients their results generally trended to turn from positive to negative in all assays. In another patient we were able to determine that his CSF inflammation (as determined by CSF leukocyte count) was declining over 4 serial samples, and all his assays in the present study also turned negative. The final patient worthy of individual mention is a patient with AIDS, as it is noteworthy that all the CSF antibody assays were positive, indicating this co-morbidity did not interfere with CSF antibody production. Over a 4-year period, the antibody tests remained positive on treatment. 

### 3.10. Rabbit CSF

Twelve rabbit study CSFs were available for testing, 4 from animals prior to infection, and 8 (half untreated controls and half treated with intravenous amphotericin B) on day 14 or 15 post-infection. With the 1-strip LFA, all 8 infected rabbit CSFs gave a strong positive test for coccidioidal antibody, with a positive control test line as well, the latter indicating (as it would in human CSF testing) a valid assay. The CSFs from the 4 uninfected rabbits also yielded a positive control test line, but no positive test line.

## 4. Discussion

The study here of 49 patient specimens, and 40 control specimens, is, to our knowledge, the largest comparative study of coccidioidal CSF diagnosis to date. Most assays studied had excellent sensitivity for the diagnosis of coccidioidal meningitis, and were efficacious in separating cases from controls (Table 6). Assays that do not require sending specimens to special reference laboratories offer logistical advantages. With the availability of kits, local hospital laboratories in endemic areas can be the source of testing. LFA assays do not even require any laboratory, are simple to use, and give rapid results; potentially even at the bedside.

Previous studies with the classical CF antibody test on CSF have reported false negative results that range from 17–41% [14,15,16]. A recent study [17] found CSF CF antibody testing to be 70% sensitive and 100% specific, virtually identical to what we found (Table 4a) with our specimens and assays. This same study reported pooled EIA results on CSF from two manufacturers (not stated), and found a sensitivity for IgM of 8%, and for IgG, 85%.

A recent addition to the CSF diagnostic armamentarium is the detection of coccidioidal antigen [17]. In CSF, a sensitivity of 93%, specificity of 100%, PPV of 100%, and NPV of 97% were the reported indices. These results are virtually the same as what we report with the 1-strip and 2-strip LFAs, and IMMY EIA assays (Table 6), although the NPV is higher. That antigen test is more rapidly performed than the classical antibody tests, but requires sending to one reference laboratory, with the ensuing turnaround time delay. The sensitivity is virtually identical to what was reported re CSF with the BDG antigen assay [7], although the other indices were higher for the coccidioidal antigen test. The BDG assay is not specific for coccidioidal antigen, but as the only other common mycosis involving the CNS is cryptococcosis (for which highly useful and widely available diagnostic methods, proven over decades [18], and newer convenient diagnostic tools [19] exist, and is a condition where BDG assay is less sensitive anyway), the lack of BDG specificity is less of a problem than one might expect. A possible convenience for BDG testing is that many hospitals have BDG kits in-house, because they are used for serum diagnosis of opportunistic mycoses in compromised hosts. There was no correlation found between coccidioidal CSF antigen tests with CSF coccidioidal antibody titers [17], as was also found in the present study between BDG antigen results and the results of the antibody tests (Table 7), and previously for CSF BDG titers and CSF CF titers [7]. Our interpretation of such findings would be that antigen-antibody binding in CSF can remove antigen and/or antibody from the CSF, and then the undetectable analytes ablate the possibility of the concordance such as seen among various antibody test results (Table 7).

The few, infrequently encountered and potentially diagnostically difficult ventricular specimens did not present problems for several of the assays in the present study.

The previously reported PCR assay study [11] reported good sensitivity for detection of coccidioidal DNA in tissue, but (although few CSFs were tested), inefficacy in PCR testing CSF. The latter was underscored in the present study, where present assays could detect coccidioidal antibody in previously PCR-negative specimens.

The ability to provide titers, beyond qualitative results, could potentially enhance the utility of several assays studied here, as higher titers might then be studied for correlation with true disease status, and those might provide further assurance in distinguishing cases. In addition, titers offer the possibility of correlating with therapeutic results, and be useful in following the course of patients. Our present results given, comparing 1:21 and 1:441 dilution results, already suggests utility of titration of these assays, and some further pilot studies with IMMY reagents reinforces this conclusion.

With respect to studies of interest using *sera*, Meridian EIA has been compared to classical assay methods, e.g., 92% sensitive and 81% specific [20]. Investigators have previously compared Meridian EIA and IMMY EIA for coccidioidal sera [21]. The Meridian EIA had more false positive tests for IgM, and was less reproducible across participating laboratories. The 1-strip LFA was, for serum, compared against CF and ID, and 98% agreement on positives reported [22]. It would be expected that BDG testing in CSF in meningitis would be more sensitive than BDG testing in coccidioidal sera [23], since the relatively closed CNS compartment does not have the elimination pathways available in the systemic circulation.

There are several shortcomings to the present study. We do not know the onset of disease for the case specimens we tested, thus which specimens (especially ones test negative) were early in the course, which were obtained later (when disease had more time to develop), and which (especially ones test negative) followed remission of disease. 

It would also have been of interest to have more serial samples from patients during their course, so as to have more data to attempt to correlate disease activity and test result. 

We lacked ID test data on some CF test negative patients, and some more might have been declared “classical test” positive if they were ID test positive. We did not have information on the level of CSF inflammation (CSF leukocytes, protein, glucose) in almost all specimens, to correlate with our test results. To date, testing sera in coccidioidal antibody and antigen assays [16,22] has suggested only histoplasmosis presents a significant cross-reaction problem, but meningitis in histoplasmosis is a rare event; more data about performance of the tests studied here in other CNS mycotic infections would still be of interest. Data-gathering addressed to these shortcomings can be derived in future studies, and inform them.

Our conservative method for LFA interpretation. utilizing multiple readers, give the reader an idea about how much variation there could be, among different readers, of a weakly positive test, although it likely increased the number of indeterminate results in this study. Inexpensive densitometry scanners exist for LFAs, and a quantitative cutoff value could be accurately determined with those instruments. Introducing these instruments as part of the LFA testing could have the downside of complicating bedside testing.

With respect to the rabbit studies, the antibody binding protein on the gold particles in LFA isn’t species-specific, so specific antibody from any species might react as would human antibody, explaining the positive test line. For the control LFA line to be positive requires enough cross-reactivity between human and rabbit antibody for there to be a reaction based on binding by the goat anti-human serum. Tests on the rabbit samples were 100% sensitive and specific, suggesting sufficient cross-reactivity with human antibody in LFA so as to be potentially useful in diagnosis of some other species. The animals for which this information could potentially be most useful would be dogs, as dogs are commonly infected in endemic regions [24], and can develop CNS disease. As this manuscript was being prepared, a paper appeared [25] on the utility of LFA testing on dog sera in coccidioidal diagnosis.

In conclusion, a number of reliable tools are available to the clinician in the diagnosis of this devastating complication. It is extremely important for clinicians to consider coccidioidal meningitis in the differential diagnosis of CNS infection, in the endemic areas and in patients with a relevant travel history, so that diagnostic tests are not delayed, and treatment instituted before devastating events ensue. The tests available have become increasingly convenient, easy to use and thus to repeat if necessary, including in sequential testing, and give rapid results.

## Figures and Tables

**Table 1:** IMMY 1-strip LFA (IgG+IgM) vs. True Disease Status.

Dilution: 1:21	Disease Status		
LFA	+	-	Total
+	40	0	40
-	2	12	14
Total	42	12	54
Sensitivity 95%, Specificity 100%, PPV 100%, NPV 86%.			

**Table 2:** IMMY 2-strip LFA (IgG ONLY) vs. True Disease Status.

Dilution: 1:21	Disease Status		
LFA IgG	+	-	Total
+	33	0	33
-	3	30	33
Total	36	30	66
Sensitivity 92%, Specificity 100%, PPV 100%, NPV 91%.			

Excluding 7 indeterminates (as defined in this study) for Cases and 4 for Controls.

**Table 3:** IMMY 2-strip LFA (IgG and IgM) vs. True Disease Status.

Dilution: 1:21	Disease Status		
LFA IgG and IgM	+	-	Total
+	34	0	34
-	3	30	33
Total	37	30	67
Sensitivity 92%, Specificity 100%, PPV 100%, NPV 91%.			

Excluding 6 indeterminates (as defined in this study) for Cases and 4 for Controls.

**Table 4:** Reference Methods, CF and ID, vs. True Disease Status.

	**a. CF Results vs. True Disease Status.**		
**1:21**	**True Disease Status**		
CF Results	+	-	Total
+	22	0	22
-	9	20	29
Total	31	20	51
PPV 100%, NPV 69%, Sensitivity 71%, Specificity 100%.			
	**b. CF/ID Results vs. True Disease Status.**		
**1:21**	**True Disease Status**		
CF/ID Results	+	-	Total
+	25	0	25
-	7	20	27
Total	32	20	52
	PPV 100%, NPV 74%, Sensitivity 78%, Specificity 100%		

There was one specimen that had an ID result reported and no CF result, therefore there is one more sample included in the table of CF + ID result compared to the CF only table.

**Table 5:** IMMY Antibody Enzyme Immunoassay (IgG and IgM) vs. True Disease Status.

Dilution: 1:21	DISEASE STATUS		
EIA IgG and IgM	+	-	Total
+	44	4	48
-	3	36	39
Total	47	40	87
Sensitivity 94%, Specificity 90%, PPV 92%, NPV 92%.			

**Table 6:** Tests in their optimal use.

	Sensitivity	Specificity	PPV	NPV
CF with ID	78%	100%	100%	74%
1-strip LFA	95%	100%	100%	86%
2-strip LFA combined *	92%	100%	100%	91%
IMMY EIA, combined *	94%	90%	92%	92%
Meridian EIA, combined *	71%	100%	100%	68%
BDG^	96%	82%	93%	90%

* Combined = assay for both IgG and IgM, either positive is scored as positive. PPV = positive predictive value. NPV = negative predictive value. LFA and EIA tests at 1:21 dilution. Indeterminate results excluded. ^from prior study.

**Table 7:** Percent agreement on positive test results.

	CF+ID	1-Strip LFA	2-Strip LFA	IMMY EIA	Meridian EIA	BDG
CF+ID	X	100	100	96	90	90
1-strip LFA	100	X	100	95	100	88
2-strip LFA	100	100	X	87	100	82
IMMY EIA	96	95	87	X	92	85
Meridian EIA	90	100	100	92	X	68
BDG	90	88	82	85	68	X

## Supplementary Materials

The following are available online at https://www.mdpi.com/2309-608X/6/3/125/s1, Figure S1: Receiver-Operator Curves, CSF testing, IMMY EIA.

## References

[1] Chiller T.M., Galgiani J.N., Stevens D.A. (2003). Coccidioidomycosis. Infect. Dis. Clin. N. Am..

[2] Dewsnup D.H., Galgiani J.N., Graybill J.R., Diaz M., Rendon A., Cloud G.A., Stevens D.A. (1996). Is it ever safe to stop azole therapy for *Coccidioides immitis* meningitis?. Ann. Intern. Med..

[3] Stevens D.A., Shatsky S.A. (2001). Intrathecal amphotericin in the management of coccidioidal meningitis. Semin. Respir. Infect..

[4] Pappagianis D., Zimmer B.L. (1990). Serology of coccidioidomycosis. Clin. Microbiol. Rev..

[5] Johnson S.M., Zimmerman C.R., Pappagianis D. (1996). Use of a recombinant *Coccidioides immitis* complement fixation antigen-chitinase in conventional serological assays. J. Clin. Microbiol..

[6] Hung C.-Y., Yu J.-J., Lehmann P.F., Cole G.T. (2001). Cloning and expression of the gene which encodes a tube precipitin antigen and wall-associated β-glucosidase of *Coccidioides immitis*. Infect. Immun..

[7] Stevens D.A., Zhang Y., Finkelman M.A., Clemons K.V., Pappagianis D., Martinez M. (2016). CSF (1,3)-beta-D-glucan testing is useful in diagnosis of coccidioidal meningitis. J. Clin. Microbiol..

[8] Donnelly J.P., Chen S.C.-A., Kauffman C.A., Verweij P.E., Groll A.H., Baddley J.W., Sorrell T.C., Clancy C.J., Lockhart S.R., Steinbach W.J. (2019). Revision and update of the consensus definitions of invasive fungal disease from the European Organization for Research and Treatment of Cancer and the Mycoses Study Group Education and Research Consortium. Clin. Infect. Dis..

[9] Richardson M.D., Warnock D.W. (1983). Enzyme-linked immunosorbent assay and its application to the serological diagnosis of fungal infection. Sabouraudia.

[10] MedCalc Statistical Software, Version 16.4.3 (2016). MedCalc Sofware bv, Ostend, Belgium. https://www.medcalc.org.

[11] Gago S., Buitrago M.J., Clemons K.V., Cuenca-Estrella M., Mirels L.F., Stevens D.A. (2014). Development and validation of a quantitative real-time PCR assay for the early diagnosis of coccidioidomycosis. Diagn. Microb. Infect. Dis..

[12] Capilla J., Clemons K.V., Stevens D.A. (2007). Efficacy of amphotericin B lipid complex in a rabbit model of coccidioidal meningitis. J. Antimicrob. Chemother..

[13] Clemons K.V., Sobel R.A., Williams P.L., Pappagianis D., Stevens D.A. (2002). Efficacy of intravenous liposomal amphotericin against coccidioidal meningitis in rabbits. Antimicrob. Agents Chemother..

[14] Bouza E., Dreyer J.S., Hewitt W.L., Meyer R.D. (1981). Coccidioidal meningitis. An analysis of thirty-one cases and review of the literature. Medicine.

[15] Mathisen G., Shelub A., Truong J., Wigen C. (2010). Coccidioidal meningitis: Clinical presentation and management in the fluconazole era. Medicine.

[16] Kassis C., Durkin M., Holbrook E., Myers R., Wheat L. (2020). Advances in diagnosis of progressive pulmonary and disseminated coccidiodiomycosis. Clin. Infect. Dis..

[17] Kassis C., Zaidi S., Kuberski T., Moran A., Gonzalez O., Hussain S., Hartmann-Manrique C., Al-Jashaami L., Chebbo A., Myers R.A. (2015). Role of Coccidioides antigen testing in the cerebrospinal fluid for the diagnosis of coccidioidal meningitis. Clin. Infect. Dis..

[18] Diaz M.R., Nguyen M.H., Heitman J., Kozel T.R., Kwon-Chung K.J., Perfect J.R., Casadevall A. (2011). Diagnostic approach based on capsular antigen capsule detection, β-glucan and DNA analysis. Cryptococcus, from Human Pathogen to Model Yeast.

[19] Tang M.W., Clemons K.V., Katzenstein D.A., Stevens D.A. (2016). The cryptococcal antigen lateral flow assay: A point-of-care diagnostic at an opportune time. Crit. Rev. Microbiol..

[20] Weiden M.A., Lundergan L.L., Blum J., Delgado K.L., Coolbaugh R., Howard R., Peng T., Pugh E., Reis N., Theis J. (1996). Detection of coccidioidal antibodies by 33-kDa spherule antigen *Coccidioides* EIA, and standard serologic tests in sera from patients evaluated for coccidioidomycosis. J. Infect Dis..

[21] Khan S., Saboulle M.A., Oubsuntia T., Heidari A., Barbian K., Goodin K., Eguchi M., McCotter O.Z., Komatsu K., Park B.J. (2019). Interlaboratory agreement of coccidioidomycosis enzyme immunoassay from two different manufacturers. Med. Mycol..

[22] (2020). Sōna Coccidioides Ab LFA IVD Instructions for Use.

[23] Thompson G.R., Bays D.J., Johnson S.M., Cohen S.H., Pappagianis D., Finkelman M.A. (2012). Serum (1,3)-D-glucan measurement in coccidioidomycosis. J. Clin. Microbiol..

[24] Shubitz L.F., Butkiewicz C.D., Dial S.M., Lindan C.P. (2005). Incidence of *Coccidioides* infection among dogs residing in a region in which the organism is endemic. J. Am. Vet. Med Assoc..

[25] Schlacks S., Vishkautsan P., Butkiewicz C., Shubitz L. (2020). Evaluation of a commercially available, point-of-care Coccidioides antibody lateral flow assay to aid in rapid diagnosis of coccidioidomycosis in dogs. Med. Mycol..

