# Supplementary material for: Comparative Study of Newer and Established Methods of Diagnosing Coccidioidal Meningitis
Source: J Fungi (Basel). 2020 Aug 4;6(3):125. doi: 10.3390/jof6030125 (PMC7558155; doi:10.3390/jof6030125)

Supplementary Figure 1

Receiver-Operator Curves, CSF testing, IMMY EIA (dotted lines are 95% confidence limits; “CAB 102” refers to IMMY EIA)

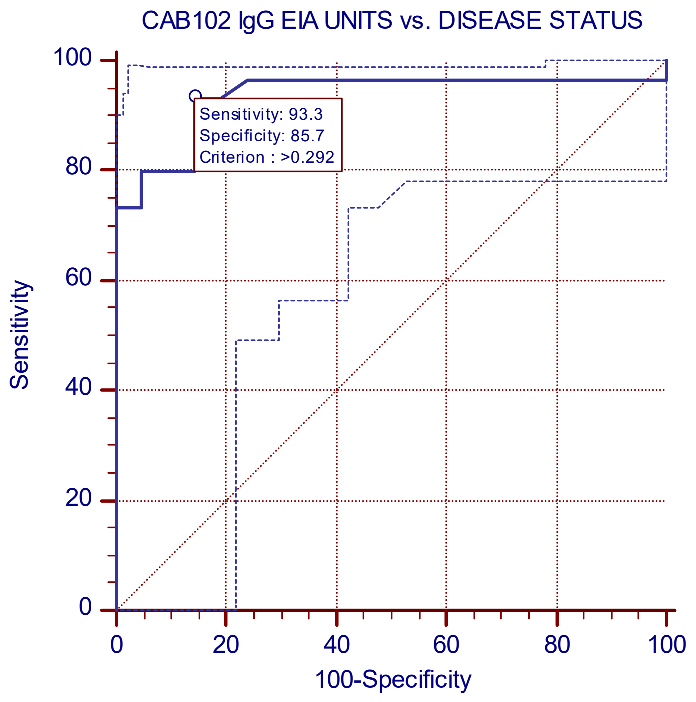

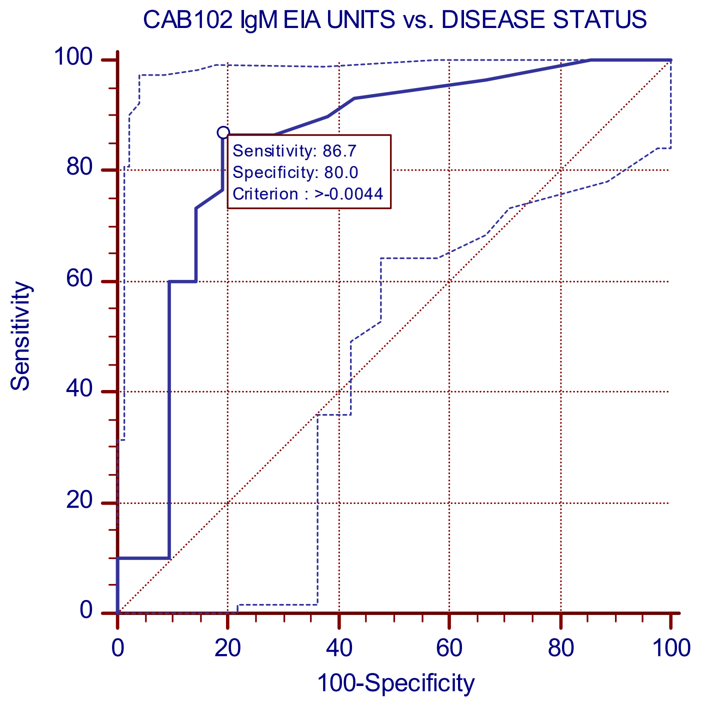

Supplement: Supplementary file 1 [file jof-06-00125-s001.pdf]
